# Secular trend in height and associated factors among adolescents in Florianópolis, Santa Catarina, Brazil, between 2007 and 2017/2018

**DOI:** 10.1590/1984-0462/2025/43/2024159

**Published:** 2025-05-02

**Authors:** Clair Costa Miranda, Jean Carlos Parmigiani De Marco, André de Araújo Pinto, Andreia Pelegrini

**Affiliations:** a Universidade de Santa Catarina, Florianópolis, SC, Brazil.; b Universidade Estadual de Roraima, Boa Vista, RR, Brazil.

**Keywords:** Growth and development, Obesity, Public health, Pediatric, Crescimento e desenvolvimento, Obesidade, Saúde pública, Pediatria

## Abstract

**Objective:**

To assess the secular trend in height among adolescents in Florianópolis between 2007 and 2017/2018, and identify factors associated with height by sex.

**Methods:**

The sample included 664 adolescents from public schools in 2007 and 1,008 in 2017/2018. Height was the dependent variable, with age, economic status, sexual maturity, physical activity, body fat (skinfold thickness), and fat-free mass as independent variables. Analysis of covariance evaluated the secular trend, and multiple linear regression identified associated factors.

**Results:**

There was a positive secular trend in height in both sexes when comparing the two surveys, with average increases of 3.5 cm in both sexes. Fat-free mass was a positive predictor and body fat was a negative predictor of height in both sexes. Additionally, physical activity emerged as a negative predictor of height specifically in boys.

**Conclusions:**

The research revealed a positive secular trend in the height of adolescents in Florianópolis. Fat-free mass contributes positively to gains in height, whereas body fat provides a negative contribution.

## RESUMO

**Objetivo:**

Avaliar a tendência secular da estatura entre adolescentes de Florianópolis entre 2007 e 2017/2018 e identificar fatores associados à estatura de acordo com o sexo.

**Métodos:**

A amostra incluiu 664 adolescentes de escolas públicas em 2007 e 1.008 em 2017/2018. A estatura foi a variável dependente, sendo idade, nível econômico, maturação sexual, atividade física, gordura corporal (dobras cutâneas) e massa livre de gordura as variáveis independentes. A análise de covariância avaliou a tendência secular e a regressão linear múltipla identificou fatores associados.

**Resultados:**

Observou-se tendência secular positiva na estatura em ambos os sexos na comparação dos dois inquéritos, com incrementos médios de 3,5 cm em ambos os dados. A massa livre de gordura foi um preditor positivo, enquanto a gordura corporal foi um preditor negativo da altura em ambos os sexos. Além disso, a atividade física surgiu como preditor negativo da altura especificamente nos meninos.

**Conclusões:**

A pesquisa revelou tendência secular positiva na altura de adolescentes de Florianópolis. A massa livre de gordura contribui positivamente para o ganho de estatura, enquanto a gordura corporal contribui negativamente.

## INTRODUCTION

Secular trends in height are globally recognized as a relevant research topic, serving as a social indicator of nutrition, health, and quality of life in specific populations.^1-3^ Whereas individual variations in height are predominantly determined by genetic factors, differences between population groups reflect the complex interplay among environmental factors, including nutrition, hygiene, health conditions, and stress.^1^ Over the past 150 years, positive trends have been reported in several regions of the world, with younger generations having higher stature than their parents.^3^ In Brazil, studies conducted in different periods (1974–1975, 1989, 2002–2003, and 2008–2009) revealed a gradual increase in the height of adolescents over the decades.^4^ However, these surveys were discontinued after 2008–2009, which limits our understanding of the growth trends of Brazilian adolescents as compared with reference populations evaluated by the World Health Organization.^5^

The gains in height observed in the Brazilian population are a reflection of improvements in socioeconomic conditions over the years, including reduced malnutrition,^6^ improved sanitation and access to treated water,^7^ advances in education,^8^ and more efficient control of infectious diseases.^7^ However, in parallel with these improvements, there was a significant increase in the rates of overweight and obesity in the Brazilian population.^6^

Although adequate nutrition plays a crucial role in determining height,^2,3^ high levels of body fat may compromise genetic growth potential,^9^ leading to early sexual maturation followed by growth stunting.^10^ However, studies have not yet investigated whether high levels of fat-free mass (FFM) contribute to enhancing height development in adolescents, which is plausible considering the endocrine and paracrine actions of muscle tissues^11,12^ and their usefulness as indicators of nutritional and behavioral conditions.^11,13,14^

Given the importance of height gains as a health indicator that is responsive to socioeconomic, nutritional, and health advances, it is essential to collect up-to-date information on height trends in adolescent populations.

This study aimed to:

1. Assess the secular trend in height among adolescents in Florianópolis, Santa Catarina, Brazil, between 2007 and 2017/2018, and
2. Identify factors associated with sex-specific height in both survey years.

## METHOD

This is an observational, epidemiological, school-based study with a repeated cross-sectional design (2007 and 2017/2018) based on secular trend analysis. The research protocol was approved by the Human Research Ethics Committees at the Federal University of Santa Catarina (protocol No. 372/2006) and Santa Catarina State University (protocol No. 2,172,699). All research procedures, including logistics, instruments, and training of the collection team, were coordinated by the same researcher in 2007 and 2017/2018.

The target population comprised adolescents of both sexes aged between 14 and 19 years who attended public high schools in Florianópolis, Santa Catarina, Brazil. The sample size was calculated from the adolescent population in 2007 (n=12,741) and 2017 (n=10,119), according to data provided by the State Secretariat of Education. Calculations followed the procedures proposed by Luiz and Magnanini,^15^ with a 95% confidence level, tolerable error of 4%, estimated prevalence of 50% for an unknown outcome, and design effect of 1.0. An additional 10% was added to the sample to account for potential losses. The minimum sample size was found to be 631 adolescents in 2007 and 624 in 2017.

The total study sample comprised 2172 adolescents, with 1146 participants from the 2007 survey and 1026 from the 2017/18 survey. In total, 315 individuals were excluded due to missing height, 89 due to missing data on sexual maturation, 67 due to incomplete responses on the socioeconomic status questionnaire, 22 due to missing skinfold measurements, and three due to incomplete physical activity data. An additional seven participants were excluded for being over 19 years old. Consequently, the final sample included 1669 adolescents, consisting of 664 from the 2007 survey and 1005 from the 2017/2018 survey.

Authorization to conduct the research in the public school system was obtained from the Florianópolis Education Management (GERED). For better representation of the reality of the city, sampling was performed in the five macro regions defined by the Municipal Health Secretariat (Mainland, Center, East, North, and South). The school with the highest number of high school students in each macro region was invited to participate in the study. Subsequently, classes were randomly selected in each school, and students were invited to participate in the research. The process was repeated until the minimum number of participants in each macro region was reached. All adolescents who agreed to voluntarily participate in the study and did not have any physical or mental condition that prevented them from completing the questionnaire or participating in the battery of physical tests were considered eligible.

All anthropometric measurements were conducted by two researchers certified at Level 1 by the International Society for the Advancement of Kinanthropometry (ISAK), with one researcher responsible for data collections in 2007 and the other in 2017/2018.

Height (dependent variable) was measured in 2007 by using a metal tape (0.1 cm resolution) attached to the wall and in 2017/2018 by using a Sanny^®^ portable stadiometer (0.1 cm resolution) (CSEP, 2021).

The independent variables comprised age (complete years), economic status, sexual maturity, and anthropometric measurements (body weight and skinfold thickness). Economic status was determined according to the Brazilian Criteria of Economic Classification, a questionnaire developed by the Brazilian Association of Research Companies (ABEP). The first survey used the 2003 version of the questionnaire. The second survey used the 2016 version, which had been updated to reflect the economic changes experienced by the population over the years.^16^ Since the two versions of the economic questionnaire employed different scoring systems, data were transformed into tertiles for analysis purposes, with the first tertile representing the lowest income level and the third tertile representing the highest income level.

Sexual maturation was self-reported based on the development of pubic hair. In 2007, the worksheets proposed by Tanner^17^ were used. In 2017/2018, an adaptation developed by Adami and Vasconcelos^18^ was employed, which features illustrations of the sexual organs to depict maturation stages rather than actual photographs. This change was intended to reduce participant discomfort and minimize visual impact. In both instruments, stage 1 represents the infantile state (prepubertal); stages 2, 3, and 4 represent the maturation process (pubertal), and stages 5 and 6 indicate a mature adult state (post-pubertal). Data on sexual maturity were categorized according to this classification. For statistical analysis, given the low participation of prepubertal adolescents (0.7%), these cases were grouped with pubertal cases, resulting in two categories, pubertal (prepubertal and pubertal) and post-pubertal.

Activity level was assessed using the International Physical Activity Questionnaire (IPAQ – short version), which has been validated for Brazilian adolescents.^19^ This questionnaire measures the number of minutes and days of moderate to vigorous physical activity (MVPA) performed during leisure time, commuting, work, and household chores over the past week. To determine the daily average of physical activity, a weighted average was calculated using the following formula: [(MVPA during the week x 5) + (MVPA during the weekend x 2)/7].

Body weight (kg) was measured using a Plenna^®^ digital scale in the 2007 survey and a Tanita^®^ digital scale in the 2017/2018 survey, both with a resolution of 100 g. Three measurements of the triceps and subscapular skinfolds were performed^20^ using a Cescorf^®^ body fat caliper (0.1 mm resolution). The quality of measurements in both surveys was assessed using the intraclass correlation coefficient (ICC 3,k), which indicated excellent reliability, with values above 0.90.^21^ Fat percentage was estimated using the equations proposed by Boileau et al.^22^ Subsequently, absolute body fat [(Body weight × Fat percentage)/100] was calculated. FFM was estimated by subtracting absolute body fat from the body weight.

The sample was characterized by descriptive statistics (mean, standard deviation, nd absolute and relative frequencies) stratified by sex. The Kolmogorov-Smirnov test was used to assess data normality, and Levene's test to verify the homogeneity of variances. Differences between survey years, stratified by sex, were analyzed using the Mann-Whitney U-test. Differences in categorical variables were investigated using the χ^2^ test.

Analysis of covariance (ANCOVA) was employed to evaluate the secular trend in height, with age, economic level, sexual maturity, physical activity, body fat percentage (%), and FFM included as covariates. The survey year (2007 and 2017/2018) was treated as a fixed factor. Results were reported as adjusted means, 95% confidence intervals (95%CI), mean differences between groups, and effect sizes calculated using eta squared (η^2^). Effect sizes were interpreted as small (η^2^<0.01), medium (η^2^ between 0.02 and 0.06), and large (η^2^>0.14). Levene's test confirmed the homogeneity of variances (p>0.05).

Multiple linear regression (Enter method) was employed to identify factors associated with height, stratified by sex. Height served as the dependent variable, while the survey year, age, economic level, sexual maturity, physical activity, FFM, and body fat percentage (%) were included as independent variables. Results were presented with non-standardized coefficients (β), standard errors, 95% confidence intervals (95%CI), standardized coefficients (β), and coefficients of determination (R^2^). The independence of residuals was assessed using the Durbin-Watson test, which yielded values of 2.19 for boys and 2.03 for girls. Multicollinearity was evaluated, with variance inflation factors (VIF) for the predictor variables remaining below 2, indicating no significant multicollinearity issues. The level of significance was set at 5%. Analyses were conducted using IBM Statistical Package for the Social Sciences (SPSS) Statistics software version 20.0.

## RESULTS

In the first survey, the sample was composed predominantly of girls (n=426, 64.2%), whereas, in the second survey, there was a greater participation of boys (n=513, 51.0%). In 2007, the majority of adolescents were in the post-pubertal phase (n=387, 58.3%), and, in 2017/2018, the majority were in the pubertal phase (n=540, 53.7%).

In comparing boys between surveys, it was observed that adolescents in 2017/2018 were older and had higher height, absolute body fat, and FFM. Furthermore, boys in the second survey had a 4,1 cm higher average height than boys in the first survey. Differences were also observed in sexual maturity (Table 1).

**Table 1:** Descriptive analysis and comparison of values between surveys, according to gender

Male		2007 (n=238) Mean±SD	2017/18 (n=513) Mean±SD	p-value*
Age (years)		16.2±1.2	16.6±1.1	<0.001
Economic status (score)		18.8±3.4	40.1±11.1	<0.001‡
Height (cm)		173.0±7.0	177.1±7.1	<0.001
Body weight (kg)		63.8±11.2	66.7±12.9	0.005
Body fat (%)		16.7±5.7	17.8±6.7	0.210
Body fat (kg)		11.1±5.7	12.5±7.0	0.049
FFM (kg)		52.8±7.1	54.3±7.8	0.006
Physical activity (min/day)		115.7±141.6	85.6±103.9	<0.001
Sexual maturation, n (%)				
	Pre-pubescent	0 (0.0)	2 (0.4)	<0.001†
	Pubescent	48 (20.2)	216 (42.1)	
	Post-pubescent	190 (79.8)	295 (57.5)	
Female		2007 (n=426) Mean±SD	2017/18 (n=492) Mean±SD	p-value*
Age (years)		16.1±1.1	16.4±1.1	<0.001
Economic status (score)		17.7±3.7	38.1±10.6	<0.001‡
Height (m)		161,3±6.2	165.6± 6.2	<0.001
Body weight (kg)		55.0±10.8	58.0±11.5	<0.001
Body fat (%)		26.5±5.5	26.6±5.7	0.711
Body fat (kg)		14.9±5.3	15.8±5.9	0.035
FFM (kg)		40.1±6.8	42.2±7.0	<0.001
Physical activity (min/day)		77.5±85.6	49.2±78.3	<0.001
Sexual maturation, n (%)				
	Pre-pubescent	0 (0.0)	10 (2.0)	<0.001†
	Pubescent	229 (53.8)	324 (65.9)	
	Post-pubescent	197 (46.2)	158 (32.1)	

* Mann-Whitney's U-test;

† χ^2^.

SD: standard deviation; FFM: fat-free mass.

‡ Differences may not reflect actual economic disparities due to varying measurement scales.

Among girls, participants of the second survey were older and had higher body weight, height, absolute body fat, and FFM. The average growth was 4.3 cm in the height of girls between the two surveys. Regarding sexual maturity, there were fewer girls in the post-pubertal phase in 2017/2018 than in 2007 (Table 1).

**Figure 1:**
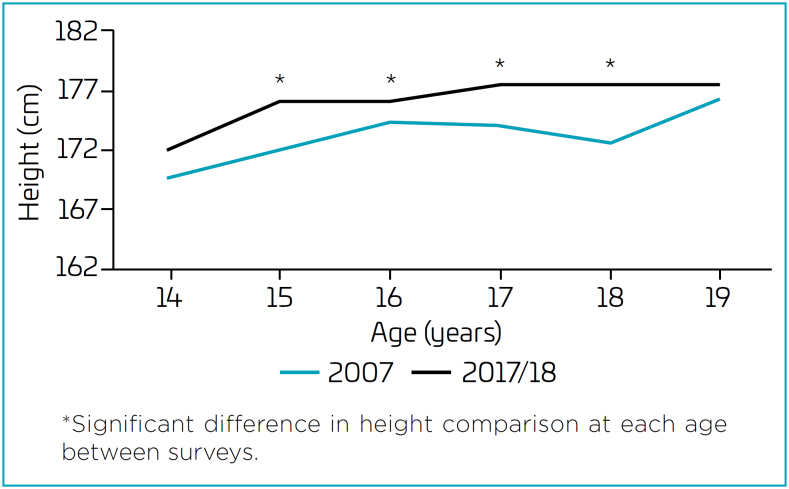
Differences in median height throughout adolescence according to the survey, by age, in boys.

**Figure 2:**
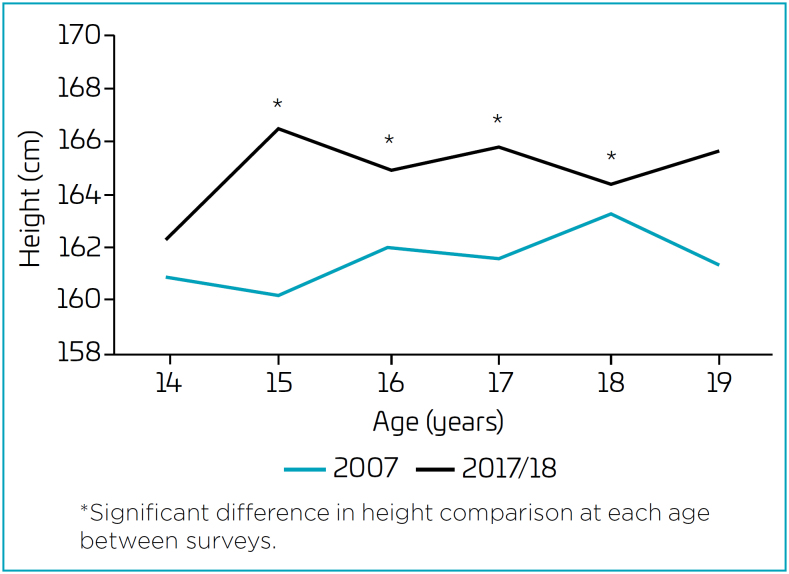
Differences in median height throughout adolescence according to the survey, by age, in girls.

Analysis of the average height per age group (Figures 1 and 2) revealed significant differences between surveys in adolescents aged 15 to 18 years. Adolescents of both sexes participating in the 2017/2018 survey had higher heights than their respective peers in the 2007 survey (Figures 1 and 2).

ANCOVA showed a positive secular trend of height in both sexes. Thus, adolescents in the second survey had higher heights than those in the first survey, regardless of age, economic level, sexual maturity, FFM and body fat (Table 2). The mean increase in adjusted height was 3.5 cm (2.00%) in boys and 3.5 cm (2.13%) in girls. These differences corresponded to a medium effect size in both sexes.

**Table 2:** Secular trend analysis of height in the sample stratified by sex.

		2007		2017/18		Δ	f	η^2^	p-value
		n	adjusted mean (95%CI)	n	adjusted mean (95%CI)				
Height									
	Male	238	173.4 (172.70–174.15)	513	176.9 (176.40–177.38)	3.5	57.52	0.07	<0.001
	Female	426	161.8 (161.23–162.27)	492	165.2 (164.72–165.68)	3.5	87.01	0.09	<0.001

Analysis of covariance (ANCOVA) adjusted for age, economic level, sexual maturity, physical activity, body fat (%) and fat-free mass.

n: absolute frequency; 95%CI: 95% confidence interval; Δ: average difference; F: ANOVA F-statistic; η^2^: Eta squared; p: significance level.

Table 3 shows the results of the regression analysis for factors associated with height, stratified by sex. For both sexes, the survey year was a positive predictor of height (p<0.001). In boys, the model explained 43.4% of the variation in height, with FFM being a positive predictor (β=0.64, p<0.001), while body fat (β=-0.22, p<0.001) and physical activity (β=-0.09, p=0.002) were negative predictors. In girls, the model accounted for 34.6% of the variation in height, with FFM as a positive predictor (β=0.50, p<0.001) and body fat as a negative predictor (β=-0.13, p<0.001).

**Table 3:** Factors associated with adolescent height, according to sex.

	Male				
	B±SE	95%CI	β	R^2^	p-value
Constant	145.53±3.25	139.10–151.91		43.4%	<0.001
Survey	3.46± 0.46	2.57–4.36	0.22		<0.001
Age	-0.29±0.19	-0.66–0.09	-0.04		0.131
Economic level	0.20±025	-0.30–0.70	0.02		0.426
Sexual maturation	0.16±0.44	-0.70–1.01	0.01		0.722
Physical activity	-0.01± 0.00	-0.01–-0.00	-0.09		0.002
Body fat (%)	-0.25±0.03	-0.32–-0.20	-0.22		<0.001
FFM	0.62±0.03	0.56–0.68	0.64		<0.001
	Female				
	B±SE	95%CI	β	R^2^	p-value
Constant	143.32±2.90	137.63–149.02		34.6%	<0.001
Survey	3.45±0.37	2.73–4.18	0.26		<0.001
Age	-0.08±0.17	-0.40–0.24	-0.01		0.629
Economic level	0.18±0.22	-0.24–0.60	0.02		0.355
Sexual maturation	0.22±0.37	-0.49–0.94	0.02		0.536
Physical activity,	0.00±0.00	-0.00–0.01	0.03		0.275
Body fat (%)	-0.15±0.03	-0.21–-0.09	-0.13		<0.001
FFM	0.47±0.03	0.42–0.52	0.50		<0.001

Model adjusted for all independent variables.

B: unstandardized coefficients; SE: standard error; 95%CI: 95% confidence interval; β: standardized coefficients; R^2^: determination coefficient; FFM: fat-free mass.

## DISCUSSION

This study revealed a positive trend in the height of adolescents over the years. There was a positive association between FFM and height and a negative association between fat percentage and height, underscoring the role of body composition in the physical growth of adolescents. These findings have substantial epidemiological implications. They suggest socioeconomic improvements in the region and highlight the need for specific interventions to promote healthy body composition. Such interventions are essential to avoid negative effects on the genetic potential of the population's stature.

A positive secular trend in adolescent height was observed over the decade, as indicated by an increase of approximately 3.5 cm in adjusted means for both sexes. This finding is in agreement with the increasing trend reported in previous studies conducted in 1974–1975 and 2008–2009 with Brazilian,^4^ Chinese,^2,10^ and Russian^23^ adolescents. In contrast, a secular trend has been observed since 1997 in the Netherlands, contrasting with the positive trends observed in other regions.^24^ One of the main hypotheses for this stagnation is that the Dutch population has already reached its genetic potential or that current lifestyle changes, such as higher-energy and less nutritious diets, could be influencing this trend.^24^

In this context, the positive secular trend observed in this study can be attributed to improvements in general health conditions and socioeconomic advances over the last decades,^6-8^ which have led to significant progress in the population´s nutritional status.^24^ This interpretation is supported by the continuous advancements in Brazil´s Human Development Index,^25^ which is closely associated with high-quality nutrition.^24^

In considering the factors associated with adolescent height, regardless of sex or survey year, we observed a negative association between fat percentage and height. The literature has shown that obese children and adolescents tend to exhibit more accelerated growth during pre-puberty. However, such differences tend to disappear during puberty, with a reduction in growth rate. By contrast, non-obese children continue to exhibit increases in height during adolescence.^26^ Despite the variations observed during growth, evidence suggests that obese individuals do not present significant changes in the expected final height.^27^ Nevertheless, obesity can compromise the genetic potential of height,^9^ as demonstrated in a previous secular trend study with Chinese children and adolescents. The referred study found that rising obesity levels may have halted population height gains.^10^ These findings highlight the importance of preventive and interventional approaches for promoting healthy eating habits and physical activity in childhood, aiming to mitigate possible negative impacts on the growth and stature development of adolescents.

Several factors may be associated with the failure to achieve the genetic potential for height due to excess body fat. Overweight young people tend to experience precocious puberty,^26,28^ a process that can influence early height gains compared with peers with lower body fat.^26^ These changes in puberty and growth can be explained by alterations in hormone and adipokine releases associated with obesity, which potentially compromise the genetic height potential of young individuals.^28^

FFM has been identified as a positive predictor of adolescent height. Previous research indicates that increased FFM during childhood is associated with greater height gains, thereby promoting linear growth.^29^ This association can be attributed to the adoption of healthy habits that enhance FFM development, such as adequate nutritional and physical activity practices. Specifically, individuals with higher FFM often exhibit better nutritional quality,^13^ highlighting the crucial role of nutrition in maintaining and building muscle mass. Adequate nutrient intake supports the balance between protein synthesis and degradation.^11^ Additionally, protein intake is vital for muscle development^29^ and serves as a positive factor for height growth in the population.^24^

Another relevant aspect is the endocrine and paracrine functions of muscle mass, including the release of myokines during muscle contractions resulting from physical exercise, which contributes to body homeostasis.^11,12^ Therefore, regular physical activity is important, not only because of the previously mentioned benefits but also for being one of the main activities responsible for increasing muscle mass.^14^ Adequate levels of physical activity throughout youth development are crucial for muscle health and good stature development. Thus, the observed negative association between physical activity and height in boys is unusual and diverges from existing literature. Given that physical activity generally supports linear growth and contributes to better muscle and bone development,^30^ this finding is particularly noteworthy.

The present study has strengths, including the use of measured data (height, weight, lean mass, and body fat percentage) rather than self-reported data, as well as validated questionnaires. Additionally, the complex sampling of the adolescent population enhances the external validity of the results.

However, some limitations must be considered:

1. The absence of nutritional data hinders a comprehensive understanding of changes in dietary patterns and their possible impact on the observed secular trend;
2. The sample was limited to adolescents from public schools, restricting the generalization of the results to those in private schools; and
3. A sample loss of approximately one-third in the 2007 survey, which, while substantial, did not significantly compromise the internal or external validity of the study, as the final sample size remained larger than the minimum required based on the cluster sampling design.

Despite these limitations, it was shown that adolescents in Florianópolis continue to benefit from the socioeconomic and health improvements observed in the region and the country. Furthermore, the identification of new potential factors associated with height gains reveals the need for a more comprehensive understanding of the determinants of this phenomenon. These aspects highlight the importance of this study for the advancement of scientific knowledge about adolescent growth. It also provides essential insights for public policies and interventions aimed at promoting improvements in the health and quality of life of adolescents.

In conclusion, adolescents in Florianópolis, Brazil, showed a positive secular trend in height, regardless of sex. This finding is a reflection of advances in socioeconomic and health conditions over the years. However, it is imperative to consider the potential impact of body composition on adolescent growth, in particular the adverse effects of body fat. Efforts aimed at promoting healthy habits of nutrition and physical activity are fundamental to ensure adequate development during adolescence.

## Data Availability

The database that originated the article is available with the corresponding author.
